# Combatting mobile colistin-resistant (MCR), metallo-β-lactamase (MBL)-producing *Klebsiella pneumoniae* persisters

**DOI:** 10.1093/jacamr/dlaf132

**Published:** 2025-07-22

**Authors:** Jack F Klem, Jan Naseer Kaur, Yang Liu, Katie Rose Boissonneault, Shivali Kapoor, Patricia N Holden, Albert Chen, Yanan Zhao, Liang Chen, Nicholas M Smith, Brian T Tsuji

**Affiliations:** Center for Infectious Diseases and Next-Generation Therapeutics, School of Pharmacy and Pharmaceutical Sciences, University at Buffalo, Buffalo, NY, USA; Division of Clinical and Translational Therapeutics, School of Pharmacy and Pharmaceutical Sciences, University at Buffalo, Buffalo, NY, USA; Center for Infectious Diseases and Next-Generation Therapeutics, School of Pharmacy and Pharmaceutical Sciences, University at Buffalo, Buffalo, NY, USA; Division of Clinical and Translational Therapeutics, School of Pharmacy and Pharmaceutical Sciences, University at Buffalo, Buffalo, NY, USA; Center for Infectious Diseases and Next-Generation Therapeutics, School of Pharmacy and Pharmaceutical Sciences, University at Buffalo, Buffalo, NY, USA; Division of Clinical and Translational Therapeutics, School of Pharmacy and Pharmaceutical Sciences, University at Buffalo, Buffalo, NY, USA; Center for Infectious Diseases and Next-Generation Therapeutics, School of Pharmacy and Pharmaceutical Sciences, University at Buffalo, Buffalo, NY, USA; Division of Clinical and Translational Therapeutics, School of Pharmacy and Pharmaceutical Sciences, University at Buffalo, Buffalo, NY, USA; Center for Infectious Diseases and Next-Generation Therapeutics, School of Pharmacy and Pharmaceutical Sciences, University at Buffalo, Buffalo, NY, USA; Division of Clinical and Translational Therapeutics, School of Pharmacy and Pharmaceutical Sciences, University at Buffalo, Buffalo, NY, USA; Center for Infectious Diseases and Next-Generation Therapeutics, School of Pharmacy and Pharmaceutical Sciences, University at Buffalo, Buffalo, NY, USA; Division of Clinical and Translational Therapeutics, School of Pharmacy and Pharmaceutical Sciences, University at Buffalo, Buffalo, NY, USA; Center for Infectious Diseases and Next-Generation Therapeutics, School of Pharmacy and Pharmaceutical Sciences, University at Buffalo, Buffalo, NY, USA; Division of Clinical and Translational Therapeutics, School of Pharmacy and Pharmaceutical Sciences, University at Buffalo, Buffalo, NY, USA; Center for Infectious Diseases and Next-Generation Therapeutics, School of Pharmacy and Pharmaceutical Sciences, University at Buffalo, Buffalo, NY, USA; Division of Clinical and Translational Therapeutics, School of Pharmacy and Pharmaceutical Sciences, University at Buffalo, Buffalo, NY, USA; Center for Infectious Diseases and Next-Generation Therapeutics, School of Pharmacy and Pharmaceutical Sciences, University at Buffalo, Buffalo, NY, USA; Division of Clinical and Translational Therapeutics, School of Pharmacy and Pharmaceutical Sciences, University at Buffalo, Buffalo, NY, USA; Center for Infectious Diseases and Next-Generation Therapeutics, School of Pharmacy and Pharmaceutical Sciences, University at Buffalo, Buffalo, NY, USA; Division of Clinical and Translational Therapeutics, School of Pharmacy and Pharmaceutical Sciences, University at Buffalo, Buffalo, NY, USA; Center for Infectious Diseases and Next-Generation Therapeutics, School of Pharmacy and Pharmaceutical Sciences, University at Buffalo, Buffalo, NY, USA; Division of Clinical and Translational Therapeutics, School of Pharmacy and Pharmaceutical Sciences, University at Buffalo, Buffalo, NY, USA

## Abstract

**Objectives:**

Therapeutic modalities for pan-drug-resistant Gram-negatives co-expressing mobile colistin resistance and metallo-β-lactamases have not been defined. Here, we devised novel strategies involving aztreonam and ceftazidime/avibactam together with polymyxin B to combat persisters of a pan-drug-resistant *Klebsiella pneumoniae* strain (*bla*_NDM-5_, *bla*_CTX-M-55_ and *mcr-1*).

**Methods:**

A hollow fibre infection model was utilized to profile the clinical combination of aztreonam + ceftazidime/avibactam + polymyxin B against a pan-drug-resistant *K. pneumoniae* clinical isolate over 168 h with reversion experiments conducted until a 216 h endpoint. The evolutionary profiles of the total and resistant subpopulations were assessed using real-time population analysis profiles. Scanning electron microscopy imaging was utilized to visualize time courses of salient structural alterations.

**Results:**

The clinical combination of aztreonam + ceftazidime/avibactam demonstrated initial activity with a total count reduction of 2.69 log_10_ CFU/mL over 24 h. Reversion experiments demonstrated complete regrowth to 10.2 log_10_ CFU/mL by 216 h. In contrast, the addition of polymyxin B to the β-lactam/β-lactamase inhibitor combination resulted in marked bactericidal activity (4.19 log_10_ CFU/mL) within 24 h and eventually below the limit of detection by 174 h. SEM imaging revealed filamentous persister cells under aztreonam selective pressure. However, with polymyxin B combinations, there was an absence of viable cells with no evidence of long filamentous persister cells, despite polymyxin resistance.

**Conclusions:**

Despite intrinsic *mcr-1* and *bla*_NDM-5_ resistance mechanisms, the proposed novel combination holds vast promise to maximally suppress the development of persisters and amplification of resistance in ‘nightmare’ pan-drug-resistant Gram-negative urgent threats.

## Introduction

Carbapenem-resistant *Enterobacteriaceae* (CRE) is classified as an ‘urgent threat’ to public health by the US Centers for Disease Control and Prevention.^1^ Clinicians have increasingly relied on polymyxins as last-line treatments, but the rise of mobile colistin resistance (MCR) complicates therapy.^2–4^ Alarmingly, *K. pneumoniae* strains co-expressing MCR and metallo-β-lactamases (MBL) have emerged, leading to pan-drug resistance (PDR) and a lack of viable treatment options.^5–9^

Bacterial persister cells represent a growing challenge in treating clinical infections as they can evade antibiotic drug pressure and give rise to new cell populations upon drug discontinuation.^10–14^ Unlike what is typically observed in antibiotic resistance, these new populations retain antibiotic susceptibility and are largely genetically unchanged. Balaban *et al.* classified persisters based on their relative metabolic activity where Type I persisters consist of pre-established dormant, stationary phase cells and Type II persisters consist of slowly growing cells that do not have a stationary phase point of origin.^11^ Regardless of the underlying mechanism of persistence, these cells pose a significant risk for treatment failure, particularly in infections caused by pathogens with existing antibiotic resistance, such as those expressing MCR and MBL.

One solution that has been proposed with success in treating patients harbouring MBL-producing Gram-negatives is the clinical combination of aztreonam, which is impervious to New Delhi metallo-β-lactamases (NDM), and ceftazidime/avibactam to inhibit existing extended spectrum β-lactamases (ESBL), such as CTX-M-55.^15^ While this β-lactam/β-lactamase inhibitor combination has shown some efficacy against MBL-producing isolates resistant to colistin,^16,17^ case reports exist demonstrating inadequate clinical responses to treatment.^18^ As we have previously shown, the PBP3 binding affinity of aztreonam can result in long filamentous persister cells in Gram-negative isolates treated with the monobactam.^19,20^ Therapeutic options for PDR Gram-negatives co-expressing MCR and MBL have not yet been defined. In this study, we explored the effect of the leading aztreonam + ceftazidime/avibactam combination together with polymyxin B. We hypothesized that despite intrinsic MCR and MBL resistance mechanisms, polymyxin combinations are a novel strategy to increase outer membrane permeability to β-lactams for mechanistic synergy by targeting long filamentous persister cells and suppressing resistance in PDR isolates.^19,21,22^ Using the hollow fibre infection model (HFIM), we evaluated a polymyxin and β-lactam/β-lactamase inhibitor combination against PDR *K. pneumoniae* (*bla*_NDM-5_, *bla*_CTX-M-55_ and *mcr-1*). Resistant subpopulations were tracked over 216 h via real-time population analysis profiles (PAPs), while scanning electron microscopy (SEM) captured structural changes over 9 days.

## Materials and methods

Minimum inhibitory concentrations (MIC) for *K. pneumoniae* S200 were determined via broth microdilution in duplicate according to CLSI guidelines.^23^ All experiments were conducted using Mueller–Hinton broth (Themor Fisher Scientific, Ref # 2275730, Becton Dickinson and Company, USA) supplemented with 12.5 mg/L Ca^2+^ and 25 mg/L Mg^2+^, and HFIM samples were plated on Mueller–Hinton II agar (MHA) (Thermo Fisher Scientific, Ref # 211438, Becton Dickinson and Company, USA). Drugs used in our analyses included aztreonam (AKSci, Ref # K379, Bristol Myers Squibb, USA), ceftazidime hydrate (Sigma-Aldrich, Ref # C3809, USA), avibactam (IHMA, Inc., Batch # G312379, USA) and polymyxin B sulphate (Sigma-Aldrich, Ref # P1004, USA).

In a HFIM, as described previously,^24^ cellulosic cartridges (C3008; FiberCell Systems, Inc., Fredrick, MD, USA) were inoculated with *K. pneumoniae* S200 (*bla*_NDM-5_, *bla*_CTX-M-55_ and *mcr-1*) to achieve an average starting inoculum of 7.57 (SD = 0.05) log_10_ colony-forming units (CFU)/mL. The system was treated with the respective antibiotic regimens over 7 days (168 h) with samples taken at intervals described below for determination of optical density at 620 nm, total counts, PAPs, pharmacokinetic validation and microscopy. Although HFIM studies show significant log_10_ fold reductions in bacterial counts during antibiotic therapy, they often overlook regrowth after antimicrobial removal.^15,19,21,25–29^ To address this, we conducted reversion experiments, discontinuing all antimicrobials after 168 h and extending the study for 48 h to a 216 h endpoint. The following regimens were simulated in the set-up design:

- Growth control
- Aztreonam [2 g prolonged infusion of 2 h in length (2hPI) every 8 h (Q8H)]
- Ceftazidime/avibactam (2/0.5 g 2hPI Q8H)
- Polymyxin B [3.33 mg/kg loading dose (LD) + 1.43 mg/kg every 12 h (Q12H)]
- Aztreonam (2hPI Q8H) + ceftazidime/avibactam (2/0.5 g 2hPI Q8H)
- Aztreonam (2hPI Q8H) + ceftazidime/avibactam (2/0.5 g 2hPI Q8H) + polymyxin B (3.33 mg/kg LD + 1.43 mg/kg Q12H)

Bacterial plate count analysis was utilized to assess the pharmacodynamics of the therapeutic regimens throughout the experiment. Pharmacokinetics replicated in the HFIM were based on previously validated run conditions, with a 2 h half-life simulated for aztreonam, ceftazidime and avibactam and an 8 h half-life simulated for polymyxin B.^21,30–34^ The longer half-life for polymyxin B required the use of supplementary doses to maintain the area under the concentration–time curve over 24 h (AUC_24_), as previously described.^21,28,31,35^ The HFIM was set up identically to a previously published and validated study using aztreonam, ceftazidime and avibactam.^15^

Bacterial counts were measured at multiple time points up to 216 h, with PAPs analysed at 0, 24, 48, 72, 120, 168, 192 and 216 h by plating diluted samples on drug-containing MHA plates. PAPs were evaluated against polymyxin B (0.5–32 mg/L), aztreonam/avibactam (2/4–32/4 mg/L) and ceftazidime (2–32 mg/L) to assess resistance profiles.

Structural changes under varying drug pressures were examined via SEM imaging at 6, 24, 168 and 216 h. Samples were fixed in 2.4% glutaraldehyde (Electron Microscopy Sciences, Ref # 16020, USA), filtered onto 0.2 µm nucleopore polycarbonate membranes (Sigma-Aldrich, Ref # WHA10417006, Whatman, USA) and dehydrated through graded ethanol washes. Following dehydration, samples were treated with hexamethyldisilazane, air-dried, gold sputter-coated and imaged using a Carl Zeiss Auriga FIB-SEM at 10 000× magnification with a 2.0 kV accelerating voltage. These analyses provided insights into the morphological responses of bacteria under antimicrobial treatment conditions.

For pharmacodynamic (PD) analysis, total bacterial count data were used to develop a mechanism-based model describing the 9-day HFIM, as previously outlined.^36^ A previously established model featuring a two-subpopulation structure was used as the starting point for model development.^37^ Model building was performed using NONMEM (version 7.5) and assisted by Perl-speaks-NONMEM (PsN, version 5.3.1) and Pirana (version 24.9.2). R (version 4.4.2) was used for data management and graphical assessment of results. Uncertainty in model parameters was calculated using sampling importance resampling (SIR) as implemented in PsN.^38^ Model evaluation was based on diagnostic goodness-of-fit plots, parameter precision, objective function values (OFVs), scientific plausibility and visual predictive checks (VPCs).

## Results

### Hollow fibre infection model

**Figure 1.**
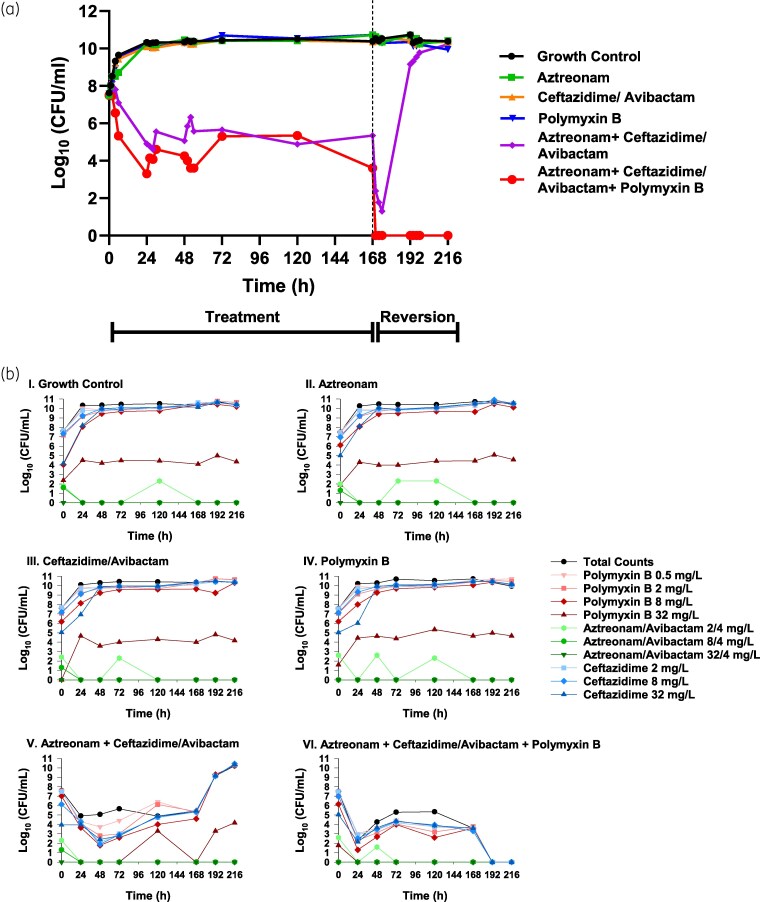
(a) Total counts: tracking total plate counts for the resistance and viability of an initial inoculum of *K. pneumoniae* S200 with a concentration of 10^7^ CFU/mL in the 9-day HFIM following exposure to various mono and combination therapies. Therapeutic regimens were simulated over 168 h. Drug administration was discontinued after the 168 h time point (dashed line), and the experiment was continued for an additional 48 h to assess population viability upon return of favourable growing conditions. (b) Population analysis profiles: total counts (black) and resistant subpopulation counts (coloured) generated from samples extracted from the regimens tested in the HFIM. Samples were plated on MHA plates imbued with arrayed concentrations of polymyxin B (red), aztreonam/avibactam (green) and ceftazidime (blue) to assess for heteroresistance.

Relevant MICs for *K. pneumoniae* S200 are listed as follows: MIC_aztreonam_ = 128 mg/L, MIC_ceftazidime/avibactam_ > 16/4 mg/L, MIC_polymyxin B_ = 4 mg/L. In our previous work, a mechanism-based pharmacodynamic model (MBM) was generated evaluating a family of NDM *Kp* isolates (*n* = 4), which included *Kp* S200. The final MBM was built, in part, utilizing the following HFIM results, and accurately described all NDM *Kp* data, with inter-experiment variability fixed to 5%CV (per cent coefficient of variation) and an estimated residual assay error of 0.87 log10 CFU/mL (<1-log sampling variability in bacterial counts).^37^ Here, growth control and monotherapy-*containing* HFIM regimens (II–IV) regrew by 24 h, increasing from an initial 7.58 log_10_ CFU/mL to a carrying capacity of 10.3 log_10_ CFU/mL (Figure 1a). In contrast, regimen V showed initial synergy, reducing the total count by 2.69 log_10_ CFU/mL over 24 h, with a seesaw pattern approaching bactericidal activity (Figure 1a). After drug removal at 168 h, regrowth to 10.2 log_10_ CFU/mL occurred by 216 h. Regimen VI, adding polymyxin B, demonstrated early enhanced synergy, reducing total counts by 4.19 log_10_ CFU/mL over 24 h. A rapid count reduction of ≥1.60 log_10_ CFU/mL to below the limit of detection was observed within 6 h following drug removal, a finding suggestive of persister dependency on drug pressure for survival during prolonged treatment. However, unlike the population reversion observed with regimen V, the total population exposed to regimen VI remained suppressed below the limit of detection through the 216 h endpoint. This suggests that the novel polymyxin combination enhances bactericidal activity and prolongs population suppression compared to regimen V alone.

### Population analysis profiles

Aztreonam/avibactam PAPs for all HFIM arms showed suppression of resistant subpopulations, with growth never exceeding 2.60 log_10_ CFU/mL at 2/4 mg/L and remaining below detectable limits at 8/4 and 32/4 mg/L (Figure 1b). Monotherapy regimens (II–IV) resembled the growth control, with resistant subpopulations exceeding 9.23 log_10_ CFU/mL within 48 h and mimicking total HFIM counts. Resistance to polymyxin B at 32 mg/L reached an average of 4.13 log_10_ CFU/mL by 72 h, never exceeding 5.35 log_10_ CFU/mL. Regimen V showed early resistance suppression, but resistance increased after 168 h, mimicking total counts. Resistance to polymyxin B at 32 mg/L was suppressed below detectable limits until 120 h and then increased to 4.18 log_10_ CFU/mL after drug removal. Regimen VI most effectively suppressed resistance, with total and resistant counts never exceeding 5.34 log_10_ CFU/mL at 24 h and below detectable limits by 192 h.

### Scanning electron microscopy

**Figure 2.**
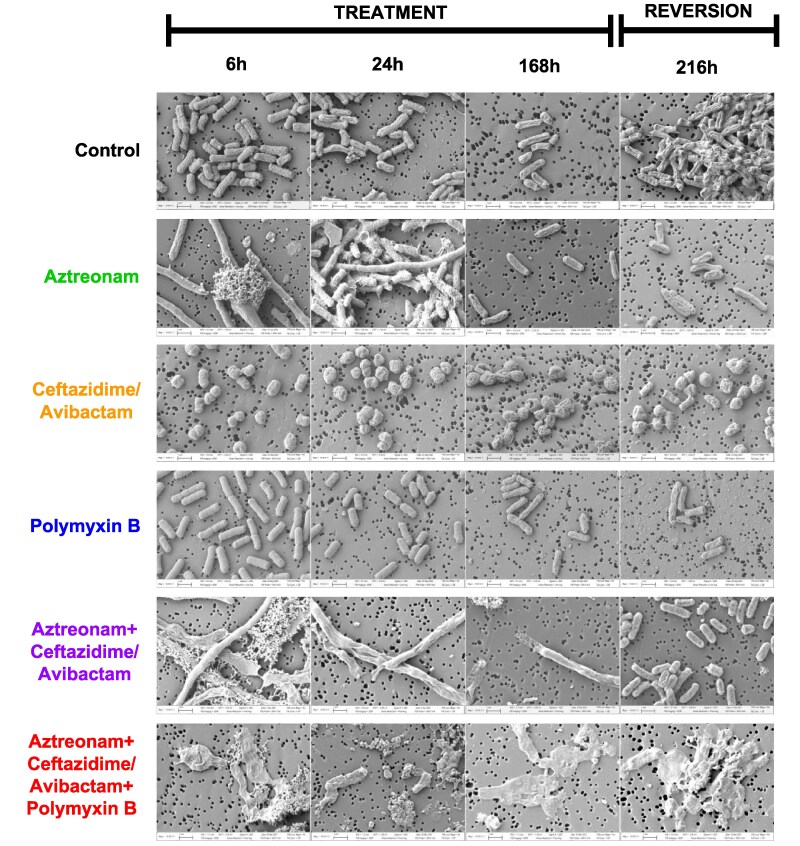
Visual representation of antibiotic response and recovery: representative scanning electron microscopy images of *K. pneumoniae* S200 HFIM at the indicated time and drug treatment as mentioned. Glutaraldehyde-fixed samples were dehydrated on 0.2 um nucleopore filters through graded ethanol washing and final hexamethyldisilazane (HMDS) addition. Samples were imaged using a Carl Zeiss Auriga Focused Ion Beam-Scanning Electron Microscope.

SEM images taken at 6, 24, 168 and 216 h from the HFIM revealed structural changes under selective antimicrobial pressures (Figure 2). Regimen I, with no drug pressure, showed no alterations, and regimen IV, where polymyxin resistance was conferred by *mcr-1*, also displayed no significant changes. Regimen III led to early formation of spheroplasts, cells with compromised cell walls.^10,39^ Regimen II, treated with aztreonam monotherapy, caused long, filamentous persister cells within 24 h, consistent with previous findings.^10^  ^,39^ These persisters were also observed in regimen V, indicating that the synergistic effect of this combination did not suppress persister formation. Later samples from both regimen II (168 h) and regimen V (216 h) showed a reversion to rod-shaped cells, suggesting the potential for persisters to revert even under continued drug exposure. However, regimen VI, which included the polymyxin combination, showed an absence of robust cells at all time points, with cellular debris predominating even at the 216 h endpoint, 48 h after drug removal. This indicates that the polymyxin combination was effective in suppressing persister formation and subsequent reversion.

### Replication–persistence–pharmacodynamics model

**Figure 3.**
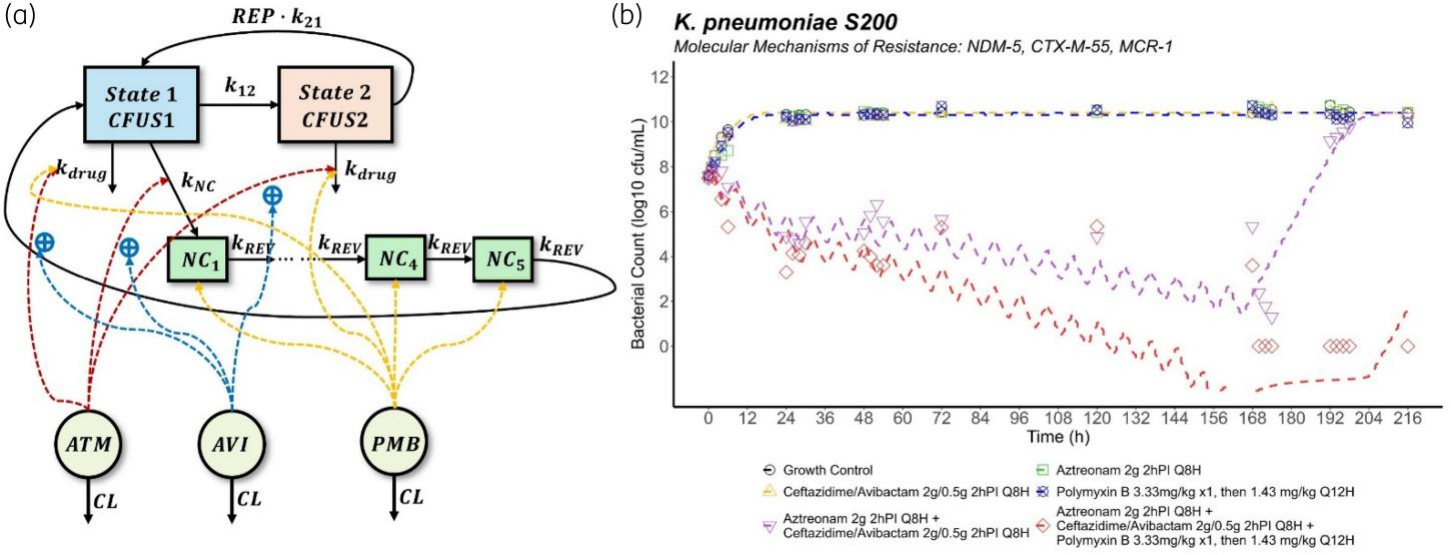
Model structure and fits: (a) schematic representation of the semi-mechanistic *replication–persistence–pharmacodynamics (RPPD) model* for different dosing regimens against *K. pneumoniae* S200 in the HFIM. State 1, bacteria in a vegetative state; State 2, bacteria in a replicative state prior to dividing; NC, non-colony-forming persisters; ATM, aztreonam; AVI, avibactam; PMB, polymyxin B. The definitions of the model parameters are presented in Table 1. (b) Fits of the developed RPPD model to the observed viable bacterial counts on a semi-logarithmic scale.

A semi-mechanistic *replication–persistence–pharmacodynamics (RPPD) model* was developed to characterize the HFIM data. A schematic representation of the final RPPD model is shown in Figure 3(a), and model parameter estimates are summarized in Table 1. The model reasonably well captured the bacterial growth and killing dynamics of the target strain under various dosing regimens in the HFIM, as illustrated in Figure 3(b). To improve model parsimony and robustness, the final model structure was simplified to a single bacterial population, given that one of the original two subpopulations had an estimated fraction close to zero with low estimation precision. A life cycle model was used to describe bacterial growth and replication in the HFIM. The replication process was simplified into two states, a vegetative state (State 1, preparing for replication) and a replicative state (State 2, prior to dividing). The transition from State 1 to State 2 was defined by a first-order growth rate constant ($k_{12}$). The replication rate constant, $k_{21}$, was assumed to be rapid (fixed to 50). β-Lactam-induced killing was modelled using Hill-type functions. In contrast, the bactericidal activity of polymyxin B was modelled using a second-order killing rate constant, reflecting prior findings that its action involves self-facilitated uptake and demonstrates a non-linear, non-saturable killing pattern *in vitro.*^21,40^ Imaging studies investigating aztreonam-based combinations against NDM-producing *K. pneumoniae* revealed the formation of filamentous cells, which are viable but non-culturable.^19,41^ This phenomenon was incorporated into the model as a transit compartment where persister cells emerged in response to aztreonam concentration. These persisters were non-replicating, remained susceptible to polymyxin B and had the potential to lead to infection relapse in the event of premature treatment discontinuation. Their behaviour was modelled using a series of transit compartments with a single transfer rate ($k_{REV}$).

**Table 1.** Model parameter estimates

Parameter	Definition	Estimate (RSE %)
**Bacterial growth parameters**		
MGT, min	Mean generation time of the population	99.7 (36.7)
LGIBMAX, log10(CFU/mL)	Maximum bacterial population size	10.4 (7.4)
LGINOC, log10(CFU/mL)	Starting inoculum	8.01 (12.9)
$K_{DIV},$ 1/h	Doubling rate constant	50 (fixed)
**Bacterial killing parameters**		
$KILL_{PMB},L/\mathrm{mg}/h$	Polymyxin B second-order killing rate constant	0.0325 (32.3)
$EMAX_{ATM},1/h$	Maximum killing rate for aztreonam	1.50 (37.6)
$EC50_{ATM},\mathrm{mg}/L$	Aztreonam concentration for 50% $EMAX_{ATM}$	64 (fixed)
$\gamma_{ATM}$	Shape parameter for aztreonam-induced killing	10 (fixed)
$IMAX_{AVI}$	Maximum reduction in $EC50_{ATM}$ by avibactam	1 (fixed)
$IC50_{AVI},\mathrm{mg}/L$	Avibactam concentration for 50% $IMAX_{AVI}$	3.01 (20.5)
$\gamma_{AVI}$	Shape parameter for avibactam-induced inhibition	10 (fixed)
**Bacterial persistence parameters**		
LGKNCMAX,log10(1/h)	Log first-order rate of persister cell formation	−2.22 (25.9)
$LGK_{REV},log10\;(1/h)$	Log first-order rate of persister cell reversion	−0.888 (20.6)
**Model residual variability**		
$\sigma_{ADD},log10\;(\mathrm{CFU}/\mathrm{mL})$	Constant residual variability on log scale	0.782 (24.0)

## Discussion

Therapeutic options for PDR Gram-negatives co-expressing MCR and MBL remain largely unexplored. This study evaluated the pharmacodynamics of novel polymyxin combinations against PDR *K. pneumoniae* (*bla*_NDM-5_, *bla*_CTX-M-55_ and *mcr-1*). A polymyxin + β-lactam/β-lactamase inhibitor combination demonstrated significant synergy and resistance suppression, as confirmed by HFIM and SEM. Cellular debris in the combination treatment arms aligned with marked CFU/mL reductions over 168 h. Notably, this regimen eradicated the bacterial population post-treatment, with SEM revealing robust rod-shaped cells in all 216 h samples except for the polymyxin-treated group, where debris dominated. The prolonged suppression is attributed to polymyxin B’s dual action—binding lipopolysaccharides and penetrating bacterial membranes.^22,28,42^

Resistance to last-line polymyxin therapies among CRE pathogens is rapidly emerging. In an Italian hospital, 93 cases of *K. pneumoniae* bloodstream infections resistant to both carbapenems and colistin were reported, accounting for 25% of cases and peaking at 41% during the outbreak.^42–45^ In the present study, resistant polymyxin B PAP counts for regimen VI followed the same trend as HFIM total plate counts, becoming undetectable by 192 h. These results suggest that the novel polymyxin combination does not amplify polymyxin B-resistant subpopulations despite pre-existing MCR resistance.^19^

Aztreonam + ceftazidime/avibactam has been used successfully against MBL-producing CRE isolates resistant to colistin.^16,17^ Here, we demonstrated early killing that persisted during treatment with aztreonam + ceftazidime/avibactam; however, population recovery was evident after therapy cessation, as observed previously.^10^ Although the early killing matched results reported by others,^17,46^ we observed the formation of long filamentous persisters, which we hypothesized was due to aztreonam exposure. Aztreonam-induced filamentation represents a unique mechanism of persistence that can be thought of as a hybrid between the Type I and Type II definitions provided by Balaban *et al*.^11^ These filaments, which do not fully divide due to PBP-3 inactivation by aztreonam, resemble a hybrid of Type I and Type II persisters.^19,20,47,48^ While they are not dormant like Type I persisters, they elongate without lateral partitioning, replicating DNA. We propose and have demonstrated that this drug-induced filamentation process leads to cellular regrowth and population proliferation in the absence of drug pressure.^19^

We previously reported aztreonam’s ability to generate long filamentous persisters in MBL strains, and here, we observed similar persister formation with aztreonam + ceftazidime/avibactam.^19^ This suggests that the therapeutic synergy of this combination does not prevent persister formation. With the recent FDA approval of aztreonam/avibactam, filamentous persister suppression has not been investigated.^49–51^ The extent of persister formation associated with aztreonam/avibactam, and its potential for synergy with polymyxin B, represents an important area of study necessary to establish its place in therapy.

Our HFIM findings, showing bacterial regrowth after drug removal, highlight the risk of reinfection post-treatment. A 2018 retrospective study by Shaw *et al*. reported clinical recurrence in one-third of patients treated with aztreonam + ceftazidime/avibactam for MBL and ESBL co-producing *K. pneumoniae*, suggesting that this regimen alone may not fully eradicate infection*s.*^18^ However, adding polymyxin B may help prevent persister formation and reversion. With rising multidrug resistance, our study supports the potential of aztreonam + ceftazidime/avibactam + polymyxin B for faster bacterial killing and persister suppression, offering promise against pan-resistant Gram-negative superbugs.

While this study focuses on developing novel therapeutic strategies against a PDR strain of *K. pneumoniae* S200, we recognize certain limitations. The HFIM experiments described here lack human cellular and immune components, necessitating future *in vivo* studies to assess toxicodynamics, host–pathogen interactions and translational potential of the regimens. Additionally, while our findings are currently specific to *K. pneumoniae*, further research is required to extend these insights to *Acinetobacter baumannii* and *Pseudomonas aeruginosa*, addressing a broader range of CDC-designated Gram-negative threats. Resistance emergence highlights the need for further studies in complex infection models. The semi-mechanistic RPPD model, simplified to a single bacterial population, may overlook resistance heterogeneity. Lastly, while SEM imaging provided structural insights, complementary molecular analyses could further elucidate persister formation and resistance mechanisms. Ongoing studies in our lab aim to bridge these gaps.
